# Extracellular Vesicles from NSC-34 MN-like Cells Transfected with Mutant SOD1 Modulate Inflammatory Status of Raw 264.7 Macrophages

**DOI:** 10.3390/genes15060735

**Published:** 2024-06-03

**Authors:** Elisabetta Carata, Marco Muci, Stefania Mariano, Simona Di Giulio, Annamaria Nigro, Alessandro Romano, Elisa Panzarini

**Affiliations:** 1 Department of Biological Sciences and Technologies, University of Salento, 73100 Lecce, Italy; elisabetta.carata@unisalento.it (E.C.); marco.muci@unisalento.it (M.M.); stefania.mariano@unisalento.it (S.M.); simona.digiulio@unisalento.it (S.D.G.); 2 IRCCS San Raffaele Scientific Institute, Division of Neuroscience, Institute of Experimental Neurology, 20132 Milan, Italy; nigro.annamaria@hsr.it (A.N.); romano.alessandro@hsr.it (A.R.)

**Keywords:** extracellular vesicles, amyotrophic lateral sclerosis, pro-inflammatory macrophages, anti-inflammatory macrophages, NSC-34 cells, Raw 264.7 macrophages, mSOD1

## Abstract

Amyotrophic lateral sclerosis (ALS) is a neurodegenerative disease targeting the brain and spinal cord. Non-neuronal cells, including macrophages, may contribute to the disruption of motor neurons (MNs), neuromuscular junction dismantling and clinical signs of ALS. Understanding the modality and the effect of MNs–macrophage communication is pivotal. Here, we focus on extracellular vesicle (EVS)-mediated communication and, in particular, we analyze the response of macrophages. NSC-34 cells transfected with mutant SOD1 (G93A, A4V, G85R, G37R) and differentiated towards MN-like cells, and Raw 264.7 macrophages are the cellular models of the study. mSOD1 NSC-34 cells release a high number of vesicles, both large-lEVs (300 nm diameter) and small-sEVs (90 nm diameter), containing inflammation-modulating molecules, and are efficiently taken up by macrophages. RT-PCR analysis of inflammation mediators demonstrated that the conditioned medium of mSOD1 NSC-34 cells polarizes Raw 264.7 macrophages towards both pro-inflammatory and anti-inflammatory phenotypes. sEVs act on macrophages in a time-dependent manner: an anti-inflammatory response mediated by TGFβ firstly starts (12 h); successively, the response shifts towards a pro-inflammation IL-1β-mediated (48 h). The response of macrophages is strictly dependent on the SOD1 mutation type. The results suggest that EVs impact physiological and behavioral macrophage processes and are of potential relevance to MN degeneration.

## 1. Introduction

The involvement of macrophages in the emergence and development of amyotrophic lateral sclerosis (ALS) as well as the role of inflammation in the exacerbation of symptoms are not still well considered. Early data suggest that neurodegenerative diseases are not considered as brain-restricted conditions and indicate that immune cells, including macrophages, are key contributors in the onset of diseases. Recent evidence suggests that immune cells can act directly from the periphery and not only after infiltrating the Central Nervous System (CNS), contributing to motor neuron injury and cell death [1,2].

Several genes have been identified as being involved in the development of ALS, e.g., C9orf72, SOD1, FUS, TARDBP, VCP, and UBQLN2, whose encoded mutated proteins not only cause ALS but also promote immune dysfunction suggesting the contribution of immune system-driven inflammation to the ALS pathogenesis [3].

Among genes involved in ALS, superoxide dismutase 1 SOD1 plays a critical role in the ignition of the inflammatory process in microglia by affecting the activation of IL-1β pro-inflammatory cytokine via the activity of caspase-1 [4]. Studies using mice carrying mutant SOD1 demonstrate that by blocking Interleukin-1ß, the inflammatory reaction is attenuated, and the animal’s life span is prolonged along with reduction of symptoms [5]. SOD1 is a key enzyme in protecting cells from oxidative stress by catalyzing the conversion of superoxide radicals into oxygen and hydrogen peroxide. In the context of ALS, mutations in the SOD1 gene have been identified as a causative factor in the development of familial forms of the disease [6]. Mutations in the SOD1 gene can lead to both loss of function and toxic gain-of-function mechanisms of the enzyme, resulting in an accumulation of toxic superoxide radicals that can damage neurons and other cells in the nervous system involving disruption of cellular functions, aggregation of misfolded proteins, and activation of inflammatory responses. Researchers are studying how to target the SOD1 pathway to potentially develop therapies for diseases associated with SOD1 mutations [7].

Monocyte-derived macrophages can infiltrate the brain, contributing to homeostasis and immune surveillance maintenance, and regulating neuroinflammation. The peculiar feature of monocytes/macrophages is to display a double phenotype, anti-inflammatory M2 or pro-inflammatory M1, that can overlap in a continuum depending on environmental signalling. The M2 phenotype is protective because it can suppress pro-inflammatory cytokine signalling; conversely, pro-inflammatory M1 one is neurotoxic by enhancing pro-inflammatory cytokine synthesis and secretion [8].

The recruitment of high numbers of monocytes to the brain and the consequent enrichment of monocyte-derived macrophages that can interfere with microglia and, in turn, exacerbate the inflammatory status in the brain characterizes ALS [9]. This interaction impacts ALS pathology that is still not well-known and defined, suggesting a potential role of inflammation in motor neuron (MN) degeneration characterizing ALS and causing clinical features of the disease, including progressive muscle weakness, loss of all muscle control, and paralysis [10,11]. In fact, during motor neuron degeneration other cells, including macrophages, in the spinal cord react to the disease and become activated, contributing to the progression of the disease in ALS transgenic mouse models [12]. Since acting on the disease progression could benefit ALS patients who can only be diagnosed when the symptoms are installed, the macrophage component could be a relevant target for therapy and so the understanding of both the modality of the communication between motor neurons and macrophages/microglia and the response of the cells represents a focus of the researchers. The crosstalk between the peripheral, CNS immune cells and MNs occurs by the mediation of both soluble molecules [13] and vesicles packaged [14]. Extracellular vesicles (EVs) as vehicles for misfolded proteins and inflammatory mediators between cells have drawn particular attention in several diseases [15] including neurological diseases [16]. EVs transport a wide type of bioactive molecules, such as antigen-presenting molecules, membrane receptors, proteins, lipids, cytokines, DNA, RNA, mRNAs, and microRNAs (miRNAs), that can influence the inflammatory state of inflammation-involved cells. Moreover, EVs are capable of crossing the blood–brain barrier (BBB) that is impaired in ALS as demonstrated in rodent models and patients; in this manner, EVs released by damaged MNs cause the recruitment of macrophages into the brain and EVs released by macrophages cause exacerbation of MNs damage [16].

In ALS mouse models, it has been demonstrated that temporal regulation of inflammation occurs: initially microglia are neuroprotective and subsequently transit to a pro-inflammatory state and become neurotoxic [17].

Our interest is therefore to study the EVs-mediated communication between MNs and macrophages in terms of the activation of macrophages. We choose NSC-34 motoneuron-like cells transfected with mutant SOD1 (G93A, A4V, G85R, G37R) as an in vitro model of MNs as they share several morphological and physiological characteristics associated with mature primary motoneurons upon differentiation. The first aspect concerns the capability of mutated SOD1 (mSOD1) NSC-34 cells to release EVs and their characterization in terms of number, type and content. The second part of our work is to value the response of Raw 264.7 murine macrophages in culture with mSOD1 NSC-34 cells derived from EVs in terms of polarization in the M1 or M2 phenotype and intracellular activated pathways.

## 2. Materials and Methods

### 2.1. NSC-34 Cell Culture and Transient Transfection

NSC-34 neuroblastoma x spinal cord cells were maintained in Dulbecco’s modified Eagle’s medium (DMEM) supplemented with 10% heat-inactivated fetal bovine serum (FBS; Cambrex, BE, East Rutherford, NJ, USA), 2 mM L-glutamine (Cambrex, BE), and 100 IU/mL penicillin/streptomycin (Sigma-Aldrich, St Louis, MO, USA). Cells were subcultured every 2–3 days.

NSC-34 cells were transiently transfected with mSOD1 cDNA by using AddGene (Watertown, MA, USA) plasmids pF146 SOD1WTAcGFP1 (Plasmid #26407), pF147 pSOD1A4VAcGFP1 (Plasmid #26408), pF148 pSOD1G37RAcGFP1 (Plasmid #26409), pF149 pSOD1G85RAcGFP1 (Plasmid #26410) and pF150 pSOD1G93AAcGFP1 (Plasmid #26411) corresponding to wild type (WT), A4V, G37R, G85R and G93A mutations, respectively. In these plasmids, the mSOD is in frame with the Green Fluorescent Protein (GFP).

A total of 35 × 10^3^ NSC-34/cm^2^ cells were incubated in OptiMEM medium (Invitrogen, Carlsbad, CA, USA) and transfected using the Lipofectamine (Invitrogen, Carlsbad, CA, USA), according to the instructions of the manufacturer. The DNA/lipofectamine ratio in the mixture was 2.5 μg/2.5 μL per well. After 24 h, the medium was replaced with DMEM High-Glucose supplemented with 10% heat-inactivated fetal bovine serum (FBS; Cambrex, BE) and 2 mM L-glutamine (Cambrex, BE). 

To assess the efficiency of transfection, GFP expression in the cells was observed at fluorescence microscopy after cell nuclei staining using 4,6-diamino-2-phenylindole (DAPI, Invitrogen, Carlsbad, CA, USA). Under our conditions, after 24 h, the transfection efficiency of NSC-34 cells was 80%.

After that, NSC-34 cells were induced to differentiate in motoneurons by incubation in the differentiation medium containing DMEM High-Glucose/Ham’s F12 (ratio 1:1), 2 mM L-glutamine (Cambrex, BE), 100 IU/mL penicillin/streptomycin (Sigma-Aldrich, St Louis, MO, USA), 10% FBS (Cambrex, BE) EVs-deprived, 1% Non-Essential Amino Acids (NEAA), 1× G418, and Retinoic Acid (RA) 1 μM for 4 days.

Differentiation was detected by evaluating specific markers by Western Blotting (synaptophysin expression), RT-PCR (choline acetyltransferase (ChAT), vesicular acetylcholine transporters (VAChT) and acetylcholinesterase (AChE) cholinergic genes) and confocal microscopy (tubulin βIII) as detailed below.

The culture medium at the end of differentiation (conditioned medium-CM) was kept for experiments detailed below.

The NSC-34 cell line has been kindly provided by Dr Alessandro Romano.

### 2.2. Experiments

The 2 × 10^6^ sEVs or lEVs were added to 3 × 10^5^ Raw 264.7 macrophages in DMEM medium. Raw 264.7 macrophages were cultured also in conditioned medium (CM) from mSOD-1 motor neurons like NSC-34 cells to assess the effects of released soluble molecules. After incubation for 24 h, cells were prepared for Western Blotting and RT-PCR analysis.

Raw 264.7 macrophages were maintained in Dulbecco’s modified Eagle’s medium (DMEM) supplemented with 10% heat-inactivated fetal bovine serum (FBS; Cambrex, BE), 2 mM L-glutamine (Cambrex, BE), and 100 IU/mL penicillin/streptomycin (Sigma-Aldrich, St Louis, MO, USA). Cells were subcultured every 2–3 days.

### 2.3. Small Extracellular Vesicles Uptake by Macrophages

The extracted small extracellular vesicles were fluorescently labelled by using PKH26 Red Fluorescent Cell (#MIDI26, Sigma-Aldrich, St Louis, MO, USA). Briefly, they were mixed with PKH26 and diluent C following the manufacturer’s instructions before incubation for 5 min at room temperature (RT). Approximately 4 μL of a PKH67 dye solution were incubated with 0.5 mL of diluent C. The vesicles and PKH67 were mixed and incubated for 5 min at RT. Then, EV-free DMEM was added to the solution (1:1), mixed and incubated for an additional 5 min at RT. Finally, EVs were centrifuged at 100,000 *g* for 70 min and rinsed with PBS to remove excess dyes. The vesicles were seeded to the Raw 264.7 macrophages for 3 h of incubation. Finally, images of the cells were captured under a laser confocal microscope (Carl Zeiss LSM 700 laser-scanning microscope, Jena, Germany) after the fixation of cells with paraformaldehyde 4% for 30 min at 4 °C and staining of the nuclei with DAPI (Invitrogen, Carlsbad, CA, USA). Image acquisition was conducted with the ZEN software (Zeiss, Jena, Germany). 

### 2.4. Isolation and Characterization of Extracellular Vesicles

Extracellular vesicles (EVs) were extracted from the conditioned medium (CM) of mSOD1 differentiated NSC-34 cells at the end of 4 days of differentiation by differential centrifugation using a Beckman Coulter Ultracentrifuge Optima XE (Beckman Coulter, Brea, CA, USA). Briefly, CM was centrifuged successively at 500 *g* (10 min, room temperature (RT)), 800 g (10 min, RT), and 2000 g (20 min, RT), to remove dead cells and aggregates. Then, the resulting supernatant was centrifuged at 20,000 *g* (20 min, 4 °C). The large vesicles-enriched pellet (lEVs) was collected, and the supernatant was filtered through 0.22 µm filters (polyethersulfone filter units, Thermo Fisher Scientific, Waltham, MA, USA). The filtered supernatant was centrifuged at 100,000 *g* (70 min, 4 °C) to collect the small vesicles-enriched pellet (sEVs).

A total of 3–5 µL of each EVs sample suspended in PBS were placed on a carbon-coated grid. The grids were observed with a TEM Hitachi 7700 (Hitachi High Technologies America Inc., Dallas, TX, USA) operating at 100 kV. For immunogold labelling, EVs in PBS were adsorbed on 200 Mesh nickel formwar-coated grids, and successively floated on drops of reactive media. Non-specific sites were coated with 1% BSA in 50 mM Tris–HCL, pH 7.4 for 10 min at RT. Antibody incubation was carried out for 2 h at 4 °C in a wet chamber with mouse monoclonal antibody raised against CD63 (# sc-365604, 1:50 dilution, Santa Cruz Biotechnology, Dallas, TX, USA) in 1% BSA, 50 mM Tris–HCL, pH 7.4. Grids were successively washed once in 50 mM Tris–HCL, pH 7.4 and pH 8.2 at RT. They were then preincubated with 1% BSA in 50 mM Tris–HCL, pH 8.2 for 10 min at RT and labelled with a goat anti-mouse IgG gold-conjugated 10 nm, (Tebu bio, Le Perray en Yvelines, France) diluted 1:80 in 1% BSA, 50 mM Tris–HCL, pH 8.2 in a wet chamber for 45 min. Grids were successively washed once in 50 mM Tris–HCL, pH 8.2 then pH 7.4 and in filtrated distilled water at RT. The grids were examined using a TEM Hitachi 7700 (Hitachi High Technologies America Inc., Dallas, TX, USA).

sEVs and lEVs were analyzed for the protein expression of specific markers CD63, Alix, Annexin I, Tsg101, Flotilin, and Calnexin by Western Blot as detailed below.

Flow cytometry analysis of EVs was performed on a CytoFLEX S flow cytometer (Beckman Coulter, Indianapolis, IN, USA). Data were analyzed using FlowJo software (version 10, Tree Star, Ashland, OR, USA). Fluorescent Megamix-Plus SSC calibration beads (BioCytex, Marseille, France) were used to optimize cytometer settings and to define the EV gate. EV samples, diluted 1:200 in PBS, were divided into 300 μL aliquots and were incubated with fluorochrome-conjugated antibodies for 15 min at room temperature. The EV detection region for analysis (vSSC/FSC) was established using control EVs expressing GFP. Large and small GFP-positive EVs were analyzed for the expression of allophycocyanin (APC)-conjugated Annexin V (BioLegend, San Diego, CA, USA) and APC-conjugated CD81, respectively. Unstained samples and EV-free antibody solutions (staining control) were used to assess the fluorescence background according to the MIFlowCyt-EV guidelines [18].

### 2.5. Electron Microscopy

Scanning electron microscopy (SEM). mSOD1 NSC-34 cells, cultured on glass coverslips (1 × 10^5^ cells/13 mm diameter coverslip), were fixed with 2.5% glutaraldehyde in 0.1 mol/L cacodylate buffer pH 7.4 for one hour at ice temperature and post-fixed with 1% OsO_4_ in the same buffer for 1 h on melting ice. After fixation, cells were dehydrated with increasing degrees of acetone (25%, 50%, 70%, 90% and 100%) followed by critical point drying (Critical Point Dryer CPD EMITECH K850; Quorum Technologies Ltd., Laughton East Sussex, UK). Stub-mounted specimens were gold-coated using a Balzers Union SCD 040 (Balzers Union, Liechtenstein) and examined under a Zeiss EVO HD 15 (Carl Zeiss, Oberkochen, Germany) scanning electron microscopy (SEM).

Transmission electron microscopy (TEM). mSOD1 NSC-34 cells were fixed with 2.5% glutaraldehyde in 0.1 mol/L cacodylate buffer pH 7.4 for 1 h at ice temperature and post-fixed with 1% OsO_4_ in the same buffer for 2 h at ice temperature. Cells were stained overnight with 5% uranyl. Cells were dehydrated with increasing degrees of ethanol (25%, 50%, 70%, 90%, and 100%) and embedded in Epoxy Spurr resin (# 4221D-1; TAAB Laboratories Equipment Ltd., Aldermaston, Berks, RG7 8NA, England). The 60 nm sections were examined under a Hitachi 7700 (Hitachi High Technologies America Inc., Dallas, TX, USA) transmission electron microscope (TEM) operating at 100 kV.

### 2.6. Immunocytochemistry

NSC-34 cells were fixed with 4% (*w*/*v*) paraformaldehyde and permeabilized in Triton X-100 0.03% in PBS. After blocking of non-specific binding sites by 3% bovine serum albumin (BSA; Sigma, Roedermark, Germany), cells were incubated in primary antibody anti-tubulin beta 3 (#MCA2047, polyclonal, 1:300; Biorad, Laboratories, Hercules, CA, USA) at 4 °C overnight.

Finally, cells were labelled with a fluorescent secondary Alexa Fluor 488-labelled anti-rabbit and anti-mouse or Alexa Fluor 594-labelled anti-mouse, anti-goat and anti-rabbit (1:500, Molecular Probes, Invitrogen, Waltham, MA, USA), for visualization. Nuclei were stained with DAPI (1:5000, Invitrogen, Carlsbad, CA, USA). Samples were mounted with Mowiol and observed at laser confocal microscopy (Carl Zeiss LSM 700 laser-scanning microscope, Jena, Germany). Image acquisition was conducted with the ZEN software (Zeiss, Jena, Germany).

### 2.7. Western Blotting Analysis

NSC-34 cells, or EVs or Raw 264.7 macrophages were lysed in ice-cold radioimmunoprecipitation assay (RIPA) buffer (ThermoFisher Scientific, Waltham, MA, USA) in agitation for 30 min. Insoluble material was pelleted by centrifugation for 30 min at 13,000 *g* at 4 °C. Supernatants were transferred to a new tube, and the protein concentration was measured by Bradford assay (Biorad, Hercules, CA, USA). Proteins (10 μg) were separated by sodium dodecylsulfate-polyacrylamide gel electrophoresis (10–12% polyacrylamide, SureCast. Acrylamide Solution, ThermoFisher Scientific, Waltham, MA, USA) and transferred to nitrocellulose membranes. The membranes were blocked with 5% non-fat dry milk in Tris-buffered saline containing 0.1% Tween 20 (TTBS) for 1 h at room temperature. Anti- CD63 (# sc-365604, 1:50 dilution, Santa Cruz Biotechnology, Dallas, TX, USA), anti-Annexin A1 (#sc-130305, 1:500 dilution; Santa Cruz Biotechnology, Dallas, TX, USA), anti-Alix (#sc-53540, 1:500 dilution; Santa Cruz Biotechnology, Dallas, TX, USA), anti-Calnexin (#sc-23954, 1:500 dilution, Santa Cruz Biotechnology, Dallas, TX, USA), anti-Flotillin-1 (#sc-74566, Santa Cruz Biotechnology, Dallas, TX, USA), anti-Tsg101 (#sc-7964, 1:500 dilution, Santa Cruz Biotechnology, Dallas, TX, USA), anti-SOD1 (#10269-1-AP, 1:5000 dilution, Proteintech, Manchester, UK), anti-Synaptophysin (#VMA00073, Biorad, Laboratories, Hercules, CA, USA), anti-Hsp90 (#MA5-15863, Invitrogen, Carlsbad, CA, USA), anti-MIF (#20415-1-AP, 1:5000 dilution, Proteintech, Manchester, UK), anti-IL-1β (#26048-1-AP, 1:5000 dilution, Proteintech, Manchester, UK), anti-TNF-α (#sc-52746, 1:500 dilution, Santa Cruz Biotechnology, Dallas, TX, USA), anti-IL-6 (#sc-65989, 1:500 dilution, Santa Cruz Biotechnology, Dallas, TX, USA), and anti-TDP43 (#12892-1-AP, 1:5000, Proteintech, Manchester, UK) diluted in TTBS in 5% non-fat dry milk. Anti β-actin (#MA1-91399, 1:2000 dilution; Invitrogen, Carlsbad, CA, USA) in TTBS in 5% non-fat dry milk was used as a control. After washing with TTBS, the membranes were incubated for 1 h at room temperature with the following horseradish peroxidase-conjugated secondary antibody diluted in TTBS in 5% non-fat dry milk: rabbit anti-mouse IgG (#G-21040, 1:5000 dilution; Invitrogen, Carlsbad, CA, USA), and goat anti-rabbit IgG (#G-21234, 1:5000 dilution; Invitrogen, Carlsbad, CA, USA). The immunoreactive bands were detected by a ChemiDoc Imaging System (Bio-Rad Laboratories, Hercules, CA, USA) using a commercial enhanced chemiluminescence (ECL) reagent (Immobilon Crescendo Western HRP substrate; Merck Millipore, Darmstadt, Germany). Clarity Max ECL-Biorad (Bio-Rad Laboratories, Hercules, CA, USA) was used for the detection of immunoreactive bands of protein content of EVs. The density of the specific bands was quantified by densitometric analysis performed by Image Lab Software TM.

### 2.8. RNA Extraction, cDNA Synthesis and Real-Time PCR

Total RNA was obtained from macrophages according to the user manual of Trizol (Invitrogen, Carlsbad, CA, USA). After isolation, the concentration of RNA was determined by Nanodrop ND-1000 Spectrophotometer (Thermo Fisher Scientific, Waltham, MA, USA). In total, 1.5 μg of RNA was converted to cDNA using a single-step SuperScriptTM IV kit (Invitrogen, Carlsbad, CA, USA) protocol. Each reaction was performed in triplicate, using mRNA levels of β-Actin for normalization. Quantitative gene expression analysis was performed using CFX ConnectTM Real-Time PCR Detection System (BIORAD, Hercules, CA, USA).

The sequences of the primers used for Real-Time PCR analysis were as follows: NFkB (forward 5′-CTG AAC CAG GGC ATA CCT GT-3′; reverse 5′-GAG AAG TCC ATG TCC GCA AT-3′); IL-1β (forward 5′-TCC ATG AGC TTT GTA CAA GG-3′; reverse 5′-GGT GCT GAT GTA CCA GTT GG-3′); TNF-α (forward 5′-GCC CAT GTT GTA GCA AAC CCT CAA-3′; reverse 5′-TGG CAC CAC CAA CTG GTT ATC TCT-3′); IL-6 (forward 5′-CCA TCT GGA TTC AAT GAG GAG AC-3′; reverse 5′-CTC TGG CTT GTT CCT CAC TAC CTC-3′); IFN-α (forward 5′-GTG AGG AAA TAC TTC CAA AGA ATC AC-3′; reverse 5′-TCT CAT GAT TTC TGC TCT GAC AA-3′); IL-4 (forward 5′-GGC TAA CAG ACA TCT TTG CTG CC-3′; reverse 5′-CAG TGT CCT TCT CAT GGT GGC T-3′); IL-10 (forward 5′-GAT GCC TTC AGC AGA GTG AA-3′; reverse 5′- GCA ACC CAG GTA ACC CTT AAA-3′); TGF-β (forward 5′-ATG CCA ACT TCT GTC TGG GG-3′; reverse 5′-GGT TGT AGA GGG CAA GGA CC-3′); ChAT (forward 5′-CCA ACC AAG CCA AGC AAT CT-3′; reverse 5′-AAG GAT AGG GGA GCA GCA ACA A-3′); VAChT (forward 5′-GCG ATG TGC TGC TTG ATG A-3′; reverse 5′-TTG ACC TAA ATG GGG AGG GTA-3′); AChE (forward 5′-ACC TTC CCT GGC TTT TCC AC-3′; reverse 5′-GCA TCC AAC ACT CCT GAC CA-3′); and β-actin (forward 5′-TGA GAG GGA AAT CGT GCG TG-3′; reverse 5′-TGC TTG CTG ATC CAC ATC TGC-3′).

### 2.9. Statistical Analysis

Data are expressed as Means ± SD. Multiple comparisons were performed by two-way ANOVA. Comparisons between the two groups were performed using a student’s *t*-test (GraphPad Prism 7 software, GraphPad Software, San Diego, CA, USA). *p* < 0.05 were considered significant.

## 3. Results

**Figure 1:**
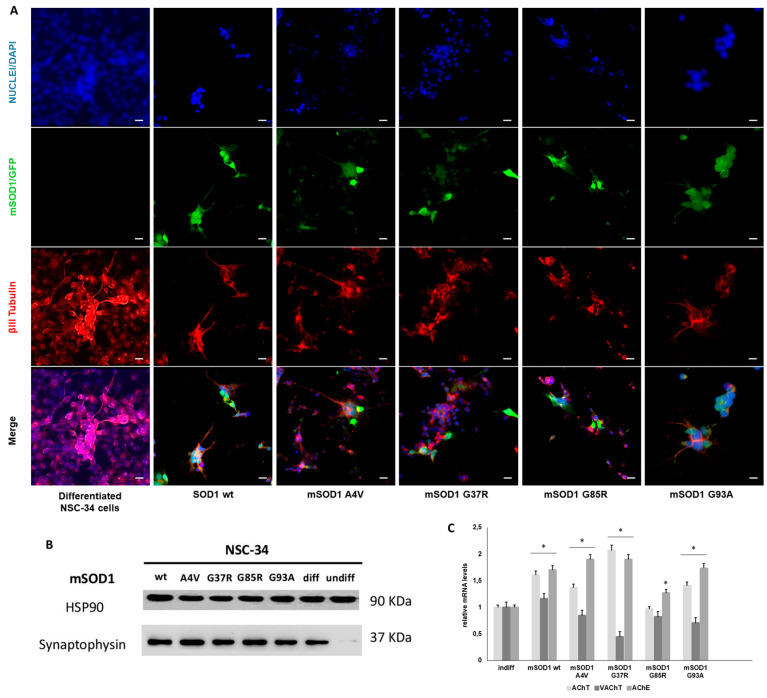
Expression of neuronal markers in NSC-34 cells during differentiation. (**A**): Confocal laser scanning microscopy images after immunolabeling with anti-βIII tubulin (red labelling, 555 laser). In green, cells transfected with plasmids with GFP (green labelling, 488 laser), in blue labelling of the nucleus with DAPI (blue labelling, 405 laser). Scale bar = 10 μm. (**B**): Representative membrane of synaptophysin protein (a marker of differentiation of NSC-34 cells towards the MN-like phenotype) expression evaluated by Western Blotting analysis. HSP90 protein was used as a control. (**C**): Relative mRNA levels of the cholinergic markers AChT (acetylcholine transporter), AChE (acetylcholinesterase) and VAChT (vesicular acetylcholine transporter) after 5 days of differentiation of NSC-34. Data are normalized to β actin and are expressed as fold of mRNA from mSOD1 differentiated NSC-34 cells vs undifferentiated NSC-34 considered as value 1. Values are means ± SD (*n* = 3); the *p*-value is from the student *t*-test (differentiated *vs* undifferentiated), (*) *p* < 0.05. Plasmids used are as follows: pF146 pSOD1WTAcGFP1 (Plasmid #26407); pF147 pSOD1A4VAcGFP1 (Plasmid #26408); pF148 pSOD1G37RAcGFP1 (Plasmid #26409); pF149 pSOD1G85RAcGFP1 (Plasmid #26410); and pF150 pSOD1G93AAcGFP1 (Plasmid #26411).

Differentiated NSC-34 cells show typical morphology with long branching processes. Neuronal maturation was confirmed by immunofluorescence staining for βIII tubulin, a specific cytoskeleton marker of motoneurons in long branching processes (Figure 1A). βIII tubulin expression correlates with the first phase of neuronal differentiation driving elongation of axons. Confocal microscopy observation shows the presence of βIII tubulin in NSC-34 cells suggesting the differentiation towards motor neuron-like phenotype. Green fluorescence due to the GFP contained in plasmids used for the transfection confirms the presence of mSOD1 in NSC-34 MN-like cells (Figure 1A). To follow whether general aspects of NSC-34 cells maturation are mirrored by the physiological maturation of cells, expression of synaptophysin and cholinergic genes implicated in ACh biosynthesis were evaluated. Synaptophysin plays several roles in motor neurons, e.g., it favors the formation of synaptic vesicles and their exocytosis and drives the synapsis formation, and biogenesis and endocytosis of synaptic vesicles. Synaptophysin expression confirmed the differentiation of NSC-34 cells as suggested by Western blotting of proteins of mSOD1 NSC-34 cells (Figure 1B). Finally, mRNA levels of choline acetyltransferase (ChAT), vesicular acetylcholine transporters (VAChT) and acetylcholinesterase (AChE) at five days of differentiation were measured (Figure 1C). ChAT into the cytoplasm synthesizes acetylcholine (Ach) that is loaded into vesicles released by the action of VAChT. AchE regulates ACh synthesis by hydrolyzing it at neuromuscular synapses. The levels of AchT and AchE are higher than the levels of undifferentiated cells; conversely, VAChT levels are always low. The levels of both cholinergic genes considered are lower than undifferentiated cells only in mSOD1G85R cells (Figure 1C).

### 3.1. Characterization of EVs

**Figure 2:**
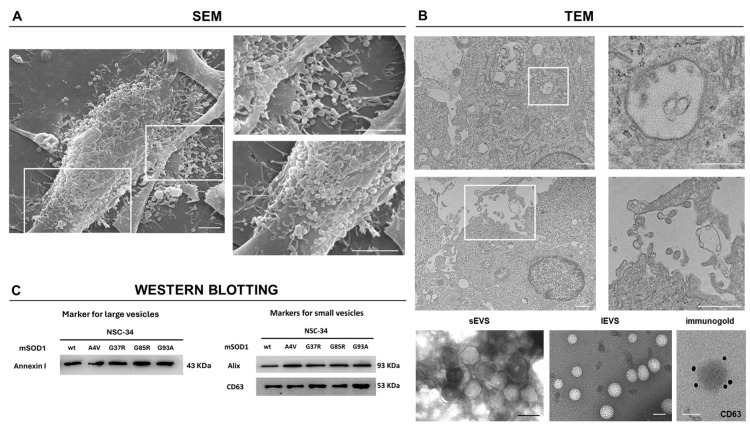
Characterization of EVs derived from mSOD1 NSC-34 MN-like cells. (**A**): Representative SEM images of the surface of G93A mSOD1 NSC-34 MN-like cells (scale bar = 3 μm). (**B**): Representative TEM images of the multivesicular bodies containing sEVs of G93A mSOD1 NSC-34 MN-like cells (scale bar = 1 μm) and TEM images of lEVs (scale bar= 300 nm) and sEVs (scale bar= 100 nm) released from G93A mSOD1 NSC-34 MN-like cells (left) and, immunogold labelling of EVs to detect CD63 at the sEVs surface (right) (scale bar = 50 nm). (**C**): Representative membrane of large and small EV-specific marker expression evaluated by Western Blotting analysis. Plasmids used for transfection: pF146 pSOD1WTAcGFP1 (Plasmid #26407); pF147 pSOD1A4VAcGFP1 (Plasmid #26408); pF148 pSOD1G37RAcGFP1 (Plasmid #26409); pF149 pSOD1G85RAcGFP1 (Plasmid #26410); and pF150 pSOD1G93AAcGFP1 (Plasmid #26411).

We first visualized the release of two EV fractions (large and small) from mSOD1 NSC-34 cells: the morphology and the distribution of large vesicles (lEVs) on the NSC-34 cells surface were analyzed by SEM (Figure 2A); conversely, as small vesicles (sEVs) are from intracellular origin, they have been imaged inside multivesicular bodies (MVBs) by TEM (Figure 2B). The central body of the mSOD1 NSC-34 cells, in addition to the filopodia, was covered by clusters of lEVs as well as sEVs that were observed within numerous MVBs visualized in mSOD1 NSC-34 cells. Furthermore, a morphological analysis of concentrated EV fraction was performed by TEM. We observe two vesicle populations: one with an average diameter of 90 nm (with a diameter ranging between 80 and 140 nm) and the other with an average diameter of 300 nm (with a variable diameter between 150 and 400 nm). In addition, there was no significant difference in the mean size of particles between different mutations considered. Independently of the diameter, the vesicles were circular shaped, and only bigger vesicles showed electron-dense material around the membrane.

Western blotting of protein extracts for characteristic extracellular vesicle markers (i.e., CD63, Alix, Annexin I, Tsg101, Flotilin, Calnexin, Hsp90) showed that only Alix and CD63, and Annexin I were visualized, confirming the presence of sEVs and lEVs in the EVs-enriched fractions (Figure 2C). Immunogold staining confirmed the presence of CD63 on sEVs surface. Both EVs fractions were negative for calnexin, an endoplasmic reticulum resident protein generally absent in EVs, confirming the validity of the chosen isolation technique.

**Figure 3:**
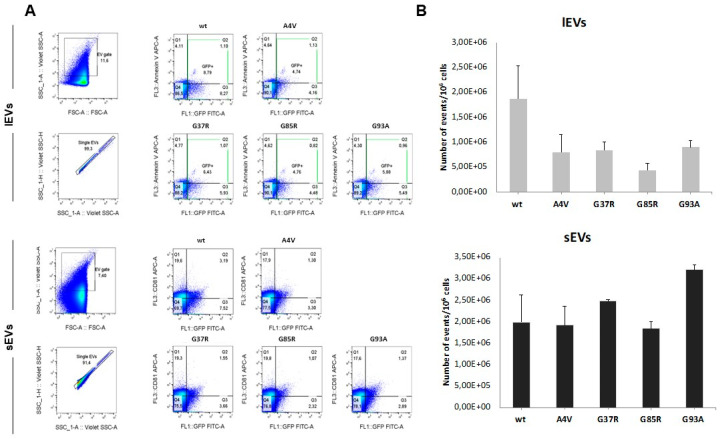
Quantitative analysis of large and small EVs isolated from mSOD1 NSC-34 MN-like cells using high-resolution flow cytometry. (**A**): Flow cytometry gating strategy for phenotyping large and small EVs. Megamix-Plus FSC is used to set FSC-vSSC for EV events. APC-Annexin V and tetraspanin APC-CD81 are used to stain isolated GFP-positive lEVs and sEVs, respectively. Before analysis on a CytoFLEX S flow cytometer, EVs are diluted 1:200 in 1X PBS. For all assays, 30 μL of the control (unstained EV) and 30 μL of each sample were measured at slow flow. (**B**), Histogram of GFP/Annexin V+ (lEVs) and GFP/CD81+ (sEVs) events in EV subtypes isolated from mSOD1 NSC-34. Graphs show mean ± SEM.

The EVs released by the differentiated NSC-34 cells expressing the wild-type and mutant human SOD1 were analyzed by flow cytometry analysis. The number of lEVs and sEVs released by the cells did not change significantly between the different conditions, and the EVs of all groups contained/carried the respective human SOD1 proteins (Figure 3).

### 3.2. Conditioned Medium of mSOD1 NSC-34 Cells Polarizes Raw 264.7 Macrophages

**Figure 4:**
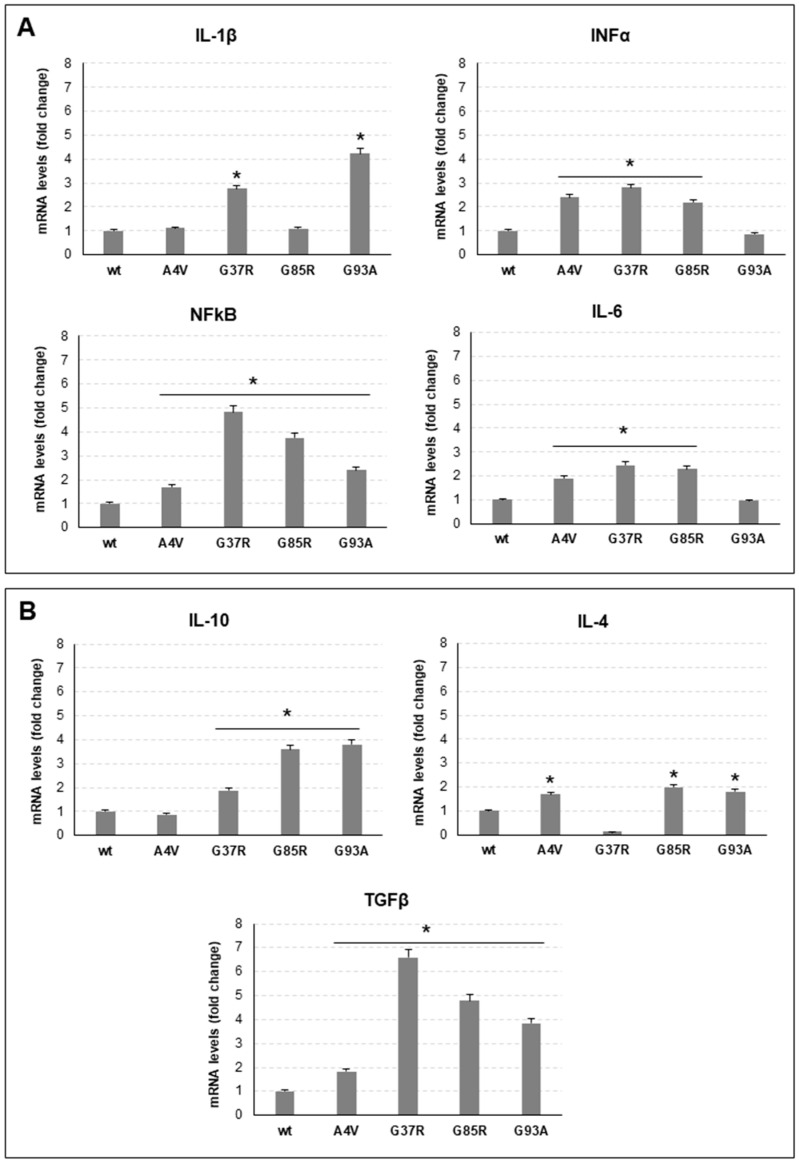
Polarization of Raw 264.7 macrophages challenged with conditioned medium (CM) of mSOD1 NSC-34 MN-like cells. (**A**): mRNA levels of pro-inflammatory interleukin 1β (IL-1β), interferon α (INFα), interleukin 6 (IL-6) and nuclear factor kappa-light-chain-enhancer of activated B cell (NFkB) in Raw 264.7 macrophages cultured 24 h in CM of mSOD1 NSC-34 MN-like cells. (**B**): mRNA levels of anti-inflammatory interleukin 10 (IL-10), interleukin 4 (IL-4), and transforming growth factor β (TFGβ) in Raw 264.7 macrophages cultured 24 h in CM of mSOD1 NSC-34 MN-like cells. Data are normalized to β actin and are expressed as fold of mRNA vs mRNA of Raw 264.7 macrophages cultured in CM of SOD1 wt NSC-34 MN-like cells, considered as value 1. Values are means ± SD (*n* = 3); *p* values are from student *t*-test (CM of mSOD1 NSC-34 MN-like cells vs CM of SOD1 NSC-34 MN-like wt cells), (*) *p* < 0.05. Plasmids used for transfection: pF146 pSOD1WTAcGFP1 (Plasmid #26407); pF147 pSOD1A4VAcGFP1 (Plasmid #26408); pF148 pSOD1G37RAcGFP1 (Plasmid #26409); pF149 pSOD1G85RAcGFP1 (Plasmid #26410); and pF150 pSOD1G93AAcGFP1 (Plasmid #26411).

To investigate the effect of mSOD1 NSC-34 in reprogramming macrophage phenotype and functions, Raw 264.7 macrophages were cultured for 24 h in the conditioned medium of NSC-34 transfected with mSOD1 plasmids and differentiated in motor neurons. Figure 4 and Table 1 report the expression levels of macrophage polarization markers after challenging with the CM of each SOD1 NSC-34 mutated cell. In general, the CM of mSOD1 NSC-34 cells induces the increase in both pro-inflammatory (Figure 4A) and anti-inflammatory (Figure 4B) marker mRNA levels. Of note is the fact that TNFα expression never changed in mSOD1 cells. Quantitative differences, which in some cases were of considerable significance, were found to be dependent on the mutation considered. Compared to NSC-34 wt, G37R mutation induces the increase in IL-1β, INFα, NFkB and IL-6, about 3-fold, 3-fold, 5-fold and 2-fold, respectively. G85R mutation induces a particularly high increase in NFkB (about 5-fold); conversely, CM of G93A mSOD1 NSC-34 cells determines in macrophages a significative increase in IL-1β transcript. Among anti-inflammatory markers, particularly high are the levels of TGFβ upon culture in CM of G37R and G85R mSDO1 NSC-34. Finally, CM of A4V mutated NSC-34 cells displays the lowest effect on polarization markers considered.

Fold numbers of increase (+) or not modified (=) compared to macrophages cultured in CM of NSC-34 SOD1 wt. The macrophage markers are categorized as pro- and anti-inflammatory. Nuclear Factor kappa-light-chain-enhancer of activated B cells (NFkB); interleukin 1 (IL-1); interleukin 6 (IL-6); interferon α (IFN α); tumor necrosis factor α (TNFα); interleukin 10 (IL-10); Transforming Growth Factor β (TGFβ); and interleukin 4 (IL-4).

### 3.3. lEVs and sEVs from mSOD1 NSC-34 MN-like Cells Regulate Polarization of Raw 264.7 Recipient Macrophages

**Figure 5:**
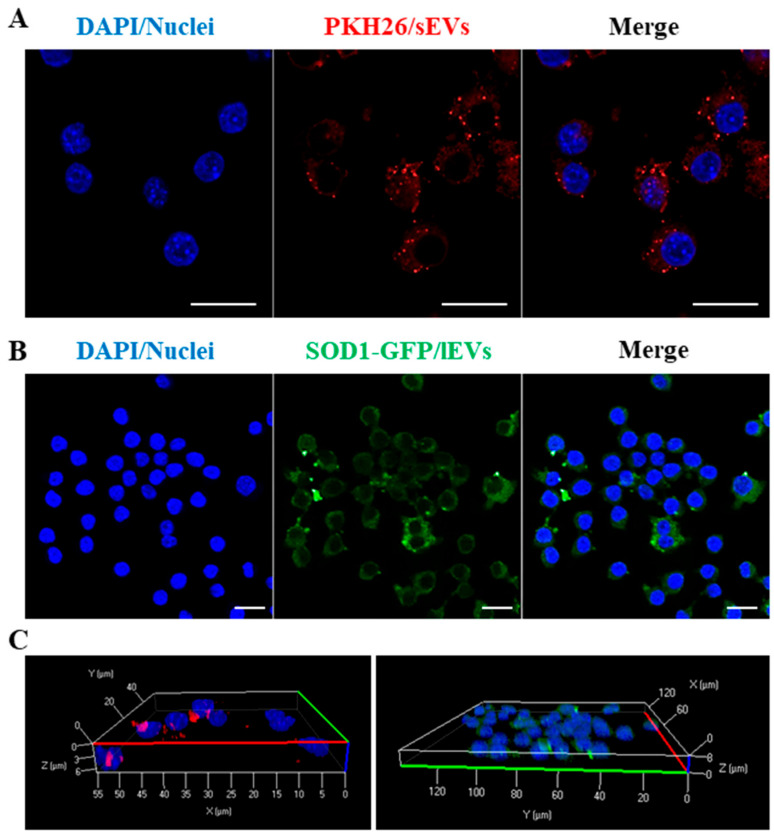
Uptake of sEVs and lEVs produced by mSOD1 NSC-34 MN-like cells by Raw 264.7 macrophages. Representative confocal images of Raw 264.7 challenged with sEVs and lEVs for 3 h. In (**A**) sEVs were labelled with PKH26 red fluorescent dye (red labelling, 555 laser); in (**B**) GFP green G93A mSOD1-derived lEVs (green labelling, 488 laser); in (**C**) 3D reconstruction of z-stack acquisition at Carl Zeiss LSM 700 confocal laser scanning microscopy. Nuclei were labelled with DAPI (blue labelling, 405 laser). Scale bar = 10 μm.

CM of cells contains both soluble and EVs packaged molecules that are pivotal in regulating cell-to-cell communication. Based on the results achieved by experiments performed with CM of mSOD1 NSC-34 cells, we decided to determine whether EVs released from mSOD1 NSC-34 MNs could affect polarization status in recipient cells. Raw 264.7 macrophages were cultured with lEVs or sEV released from mSOD1 NSC-34 MNs. First, we analyzed, by confocal microscopy, whether the sEVs and lEVs are internalized by macrophages. To this end, sEVs were firstly labelled with the lipophilic dye PKH26 as detailed in the Materials and Methods section, while lEVs are green because they contain mSOD1-GFP labelled. The PKH26-labeled sEVs and lEVs were added separately to recipient Raw 264.7 macrophages, and the preparations were incubated for 3 h. Images show that both PKH26-positive (Figure 5A) and GFP-positive (Figure 5B) vesicles were internalized by macrophages, as evidenced by the red or green puncta observed in the cells stained with DAPI. On the other hand, no signal was found in cells alone or incubated with free PKH26. Importantly, the internalization of EVs by macrophages was confirmed by 3D reconstruction (Figure 5C).

**Figure 6:**
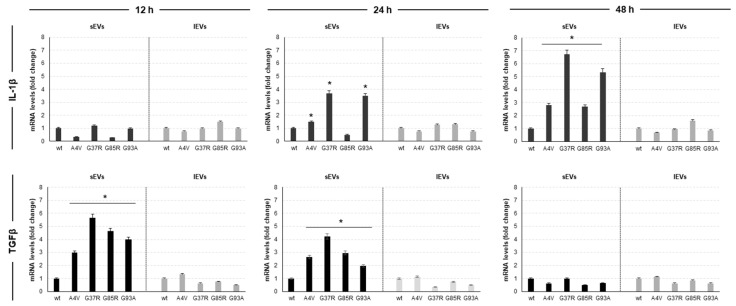
Polarization of Raw 264.7 macrophages challenged with sEVs and lEVs produced by mSOD1 NSC-34 MN-like cells. mRNA levels of pro-inflammatory interleukin 1β (Il-1β) and anti-inflammatory transforming growth factor β (TFGβ) in Raw 264.7 macrophages cultured 12, 24 and 48 h with sEVs and lEVs of mSOD1 NSC-34 MN-like cells. Data are normalized to β actin and are expressed as fold of mRNA vs mRNA levels of Raw 264.7 cultured with sEVs and lEVs of SOD1 wt NSC-34 MN-like cells, considered as value 1. Values are means ± SD (*n* = 3); *p* values are from Student’s *t*-test (sEVs and lEVs of mSOD1 NSC-34 MN-like cells vs sEVs and lEVs of SOD1 NSC-34 MN-like wt cells), (*) *p* < 0.05. Plasmids used for transfection: pF146 pSOD1WTAcGFP1 (Plasmid #26407); pF147 pSOD1A4VAcGFP1 (Plasmid #26408); pF148 pSOD1G37RAcGFP1 (Plasmid #26409); pF149 pSOD1G85RAcGFP1 (Plasmid #26410); and pF150 pSOD1G93AAcGFP1 (Plasmid #26411).

We chose to analyze the levels of transcripts of IL-1β and TGFβ at 12, 24 and 48 h of culture in the presence of lEVs and sEVs as these cytokines are the main cytokines involved in the modulation of the inflammatory status of macrophages (Figure 4). The analysis of the data obtained (Figure 6) clearly revealed three aspects: (1) the ability of the EVs produced by mSOD1 NSC-34 cells to induce the polarization of Raw 264.7 macrophages in a time-dependent manner towards both M1 and M2 phenotype; (2) the effect of EVs in terms of levels of transcripts depends on the SOD1 mutation; and (3) only sEVs elicit an effect on the polarization of Raw 264.7 macrophages. The response of macrophages to sEVs produced by mSOD1 NSC-34 cells starts as an anti-inflammatory polarization: after the treatment for 12 h with NSC-34-derived EVs, the recipient macrophages belonged to the M2 category. In fact, the relative levels of TGFβ are very high and quantitative differences were found to be dependent on the mutation considered, whereas any change in IL-1β transcripts level has been measured. Compared to sEVs isolated from NSC-34 wt, those derived from A4V, G37R, G85R, and G93A induce in Raw 264.7 macrophages an increase in TGFβ mRNA levels about 3-fold, 6-fold, 5-fold, and 4-fold respectively. At 24 h of treatment with sEVs, we noted that the recipient Raw 264.7 macrophages display both an M1 and M2 phenotype. We measured an increase in levels of IL-1β only in the culture in the presence of NSC-34 A4V, G37R and G93A mSOD1-derived sEVs. In particular, G37R and G93A mSOD1-derived EVs induce an increase in IL-1β mRNA about 4-fold. In parallel, we noted a decrease in TGFβ transcript levels about 20%, 30%, and 50% with respect to 12 h of treatment when Raw 264.7 macrophages were challenged with sEVs released by G37R, G85R, and G93A mSOD1 NSC-34 cells, respectively. Finally, at 48 h of treatment with sEVs, we do not detect mRNA levels of TGFβ whereas an increase in IL-1β transcripts has been measured. When Raw 264.7 macrophages were challenged with sEVs isolated from the culture medium of G37R and G93A mSOD1 NSC-34 cells, the IL-1β transcripts increased about 70% with respect to those measured at 24 h of treatment (Figure 6).

### 3.4. EVs Signature

**Figure 7:**
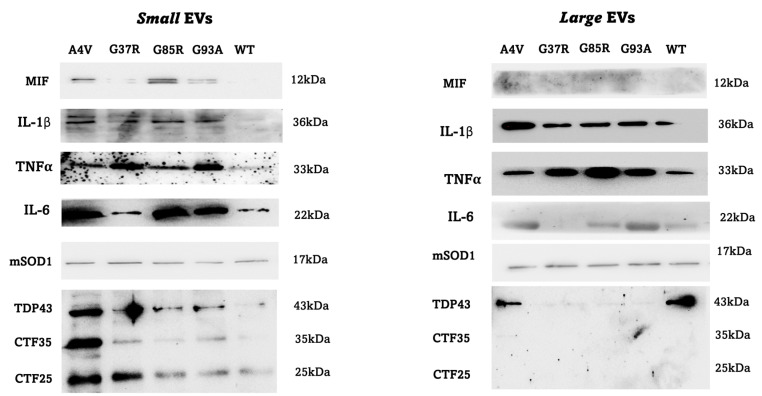
Representative membrane of protein content evaluated by Western Blotting analysis in small and large extracellular vesicles released by mSOD1 NSC-34 MN-like cells at the end of 4 days of differentiation. MIF: macrophage migration inhibitory factor, IL-1β: Interleukin 1β, TNFα: tumor necrosis factor α, IL-6: Interleukin 6, mSOD1, and TDP43: TAR DNA binding protein 43.

To understand whether the effect on the levels of transcripts of inflammatory mediators detected in Raw 264.7 macrophages is mediated by the molecules present within the EVs, different types of molecules were searched by Western blotting analysis: three pro-inflammatory cytokines (IL-1β, IL-6 and TNFα), a chemoattractant factor (MIF, Macrophage migration inhibitory factor), the aggregates of the mutated TDP43 protein and the SOD1 protein (Figure 7). In small EVs, all molecules considered are present, and the abundance of the MIF factor, IL-6 and aggregates of the mutated TDP43 protein are particularly evident. On the contrary, the MIF factor is not present in large EVs, while the abundance of IL-1β and TNF-α is high. The small EVs isolated from NSC-34 transfected with the pF147 plasmid pSOD1A4VAcGFP1 carry all the molecules considered.

## 4. Discussion

Two aspects characterize the complexity of amyotrophic lateral sclerosis (ALS): neurodegeneration involves both brain and spinal cord motor neurons (MNs), and both neurons and non-neuronal cells drive ALS pathology. Among non-neural cells, researchers are starting to consider macrophages as key players in neuromuscular junction dismantling that cause clinical signs of ALS, such as chronic and progressive muscle weakness, loss of all muscle control, and fatal paralysis, resulting in patient death [11]. This issue is based on the peculiarity of MNs to possess soma located in the central nervous system (CNS) and exposed to microglia and axons located outside the CNS and exposed to peripheral macrophages, which can also infiltrate the CNS upon MNs recruitment and interfere with microglia in modulating inflammation in the brain microenvironment [19]. Both animal and ALS patient studies have demonstrated the central role of inflammation in the occurrence and aggravating of MNs degeneration [20]. The fatal communication between immune cells, both glial cells and macrophages, and MNs in determining inflammatory status involves transcellular signalling through chemical molecules. More and more evidence indicates extracellular vesicles as a new and promising way of cell-to-cell communication in the CNS [21]. Unfortunately, to date, very little data on the role of peripheral macrophages in contributing to MNs degeneration is present, whereas the majority of research focuses on the role of microglia. In addition, the research about the role of EVs in mediating MNs-macrophages communication and, in turn, in modulating inflammation is still at infancy.

Here, we demonstrated for the first time the effects of transfected mSOD1 NSC-34 MN-derived EVs on the Raw 264.7 macrophages’ activation profile and microenvironment. Macrophages tendentially acquire an M2b activation that sustains a both protective and pathogenic environment. Data on the potential role of the mutation type on the effects mediated by EVs were also reported.

We preliminarily establish a transfection and differentiation protocol for the motoneuron-like cell line NSC-34 to investigate the EVs effects in relation to the SOD1 mutation present. NSC-34 cells developed by Cashman et al. in 1992 [22] met typical characteristics of motoneurons and were chosen as an experimental cell model to study motoneuron-like behavior in the context of ALS since the pathology can be created in this cell line [22,23]. Moreover, NSC-34 cells are also used in studies concerning the role of EVs in the spreading of ALS [24].

Here, before the differentiation, we inserted in NSC-34 cells SOD1 mutated genes by transient transfection protocol; in particular, we used four different SOD1 mutations, i.e., A4V, G37R, G85R, and G93A. Approximately 40–55% of amyotrophic lateral sclerosis (ALS) cases are attributed to mutations in over 50 identified ALS-associated genes; among these, pathogenic mutations in SOD1, C9ORF72, FUS, and TARDBP are the most prevalent. [25]. Clinical and basic research data suggest multiple causes of ALS: up to 10% of ALS cases are familial ALS (fALS) (at least one other family member is affected), the remaining 90–95% of ALS cases are sporadic ALS (sALS) (no family history). Superoxide Dismutase 1 (SOD1) codes a 153 amino acid metalloenzyme providing an important antioxidant defense mechanism against cellular respiration-derived ROS species. The mutation of SOD1 affects approximately 2% of the 168 thousand cases of ALS estimated globally; in Europe, 14.8% of fALS patients and 1.2% of sALS one show SOD1 mutation [25]. In general, the average life expectancy mSOD1 patients is extremely variable and, in some cases, does not exceed one year. More than 185 disease-associated mutations in the SOD1 gene have been identified and associated with varying disease duration and severity; for instance, patients carrying the A4V, H43R, L84V, G85R, N86S, and G93A variants exhibit rapid development of pathological conditions and shorter survival times. [25]. In April 2023, the U.S. Food and Drug Administration (FDA) granted approval for the antisense oligonucleotide tofersen for the treatment of SOD1-ALS.

Under our conditions, the transfection efficiency of NSC-34 cells was 80%. Neuronal maturation was confirmed by immunofluorescence staining for the specific cytoskeleton marker of motoneurons in long branching processes βIII tubulin, whose expression correlates with the first phase of neuronal differentiation driving elongation of axons suggesting the differentiation towards motor neurons-like phenotype. Physiological maturation of mSOD1 NSC-34 MNs-like cells has been confirmed by the expression of synaptophysin and cholinergic genes implicated in ACh biosynthesis. In neuronal differentiation, there is a distinctive morphological advancement involving neurite growth and dendritic plasticity, which is evidenced by the presence of microtubule-associated proteins. Simultaneously, there are functional alterations such as synapse formation and maintenance of neurotransmitter balance. Co-expression of enzymes responsible for acetylcholine (ACh) synthesis, storage, and breakdown is pivotal for the optimal performance of cholinergic neurons. In our hands, after five days of differentiation, AchT and AchE levels were significantly up-regulated, whereas the expression of VAchT remained unaltered; it is plausible that the absence of regulatory mechanisms governing VAchT expression could contribute to the lack of functional synapses or the formation of developing synaptic structures in an in vitro model of MNs.

In line with the burst received by EVs research in neurodegenerative diseases, including ALS, we focused our research on the understanding if our mSOD1 NSC-34 MNs-like cell model releases EVs. EVs are nowadays accepted as a way to transport biologically active molecules that are able to modulate the physiology of recipient cells, and not a simple cell disposal route with no biological significance, also in ALS.

Exosomes are considered key mediators of neuroinflammation [26], and also in ALS, exosomes can transfer mSOD1 from astrocytes to MNs contributing to the propagation of mutated SOD1 [27] as in recipient cells, mSOD1 affects the misfolding of normal SOD1 [28]. Gomes and coworkers also demonstrate the secretion of SOD1 through exosomes released by stable mouse motor neuron-like NSC-34 cells overexpressing human SOD1 wild-type and mutant SOD1(G93A) [29]. Spinal and cortical astrocytes from SOD1G93A (mSOD1) 7-day-old mice release EVs able to induce dysfunction in mSOD1 NSC-34 MNs [30]. Exosomes from NSC-34 cells mutant SOD1(G93A) transfer mSOD1 to N9 microglia cells [24].

We first analyzed small (sEVs) and large (lEVs) EVs released by mSOD1 NSC-34 MNs-like cells separately. We initially fractionated EVs based on their size into two distinct groups before conducting a comprehensive characterization encompassing quantity, size, and molecular composition. Notably, we employed mSOD1 NSC-34-derived sEVs and lEVs separately for the first time across all experimental procedures. Our findings demonstrate that all four SOD1 mutated NSC-34 cell lines consistently released a substantial quantity of EVs. Moreover, differences in size, concentration, and molecular composition were discernible between the sEVs and lEVs. Electron microscopy allowed us to observe two vesicle populations: one with an average diameter of 90 nm and the other with an average diameter of 300, and no significant difference in the mean size of particles between different mutations considered has been observed as well as independently of diameter and mutation type, the vesicles were circular shaped, and only bigger vesicles showed electron-dense material around the membrane. Flow cytometry analysis showed that the number of lEVs and sEVs released by the cells did not change significantly between the different mSOD1 types, and the EVs of all groups contained/carried the respective human SOD1 proteins. Molecular profile analysis by Western Blotting suggested a difference between lEVs and sEVs: in small EVs, all molecules considered in this study (pro-inflammatory cytokines IL-1β, IL-6 and TNFα, the chemoattractant factor MIF-Macrophage migration inhibitory factor, the aggregates of the mutated TDP43 protein) are present, and the abundance of the MIF factor, IL-6 and aggregates of the mutated TDP43 protein are particularly evident. On the contrary, the MIF factor is not present in large EVs, while the abundance of IL-1β and TNF-α is high. The small EVs isolated from NSC-34 transfected with the pF147 plasmid pSOD1A4VAcGFP1 carry all the molecules considered. The presence of mutated TDP43 protein in vesicles is particularly interesting, as it both implies and corroborates the limited and contentious findings in the literature regarding the connection between mutant SOD1 and TDP-43 modification. Additionally, it sheds light on the association between uncorrected TDP-43 levels and neuronal cytotoxicity in SOD1 ALS. TDP-43 is a nuclear protein that plays an important role in regulating RNA splicing, stability, and transport [31]. In ALS neurons, TDP-43 is fragmented in the cytoplasm and thus, prone to aggregation [32]. The majority of studies have been achieved by using mice and clinical data of ALS patients. Only a minority of fALS cases linked to SOD1 mutations exhibit cytoplasmic inclusions of TDP-43 [33]. Robertson et al. [34] demonstrated the mislocation of TDP-43 to the cytoplasm, its connection with mutated SOD1 protein, and the consequent motor neuron damage in the spinal cord of ALS mice expressing SOD1G93A [35,36]. Utilizing animal models, human tissue samples, and cell models, Jeon illustrated an increase in TDP-43 C-terminal fragments and phosphorylation levels in both motor neurons and glia of SOD1 mice and ALS patients with the SOD1G85S mutation. Furthermore, cytoplasmic localization of TDP-43 was also noted in iPSC-derived motor neurons from an ALS patient with the SOD1G17S mutation. Mutant SOD1 overexpression affects the solubility/insolubility of TDP-43 and causes motor neuron apoptotic death [37].

Following the characterization of sEVs and lEVs, we explored, to our knowledge for the first time, the modulation of macrophage phenotype in vitro induced by the EV subtypes released by SOD1-mutated MNs. Most existing studies predominantly focus on the activation of microglial cells. These studies suggest the modulation of polarization status of microglia: Pinto documented a transition in microglial phenotype from the classic M1 activated state to a blend of microglial subtypes, encompassing M2 polarized cells, and this effect is due to miRNAs present in exosomes that interfere with the metabolism of N9 microglia cells [24].

The analysis of the data obtained clearly revealed six aspects: (1) conditioned medium of mSOD1 NSC-34 cells induces in Raw 264.7 macrophages the increase in both pro-inflammatory and anti-inflammatory molecules expression; (2) TNFα expression never changed in mSOD1 cells; (3) quantitative variances, some of which were notably significant, were determined to be contingent upon the specific mutation under examination; (4) Raw 264.7 macrophages internalize both large and small EVs; (5) the ability of the EVs to induce the polarization of Raw 264.7 macrophages in a time-dependent manner towards both M1 and M2 phenotype; and (6) only sEVs elicit an effect on the polarization of Raw 264.7 macrophages. 

We observe that the response of macrophages to sEVs produced by mSOD1 NSC-34 cells starts (after 12 h of treatment) as an anti-inflammatory polarization by up-regulation of TGFβ and ends (after 48 h of treatment) as a pro-inflammatory polarization by up-regulation of IL-1β. In the middle (24 h of treatment with sEVs), the recipient Raw 264.7 macrophages display both the M1 and M2 phenotype. This suggests a complex network of signaling. However, in our hands, it is not completely clear if the effect could be exerted also by soluble molecules released by MNs.

Overall, the achieved results indicate that EVs released from mSOD1 NSC-34 MNs are internalized by Raw 264.7 macrophages, eliciting a distinctive pattern of cellular activation and suggesting that also macrophages could be players in ALS pathophysiology.

## 5. Conclusions

The results reported in this paper confirm that the EVs/macrophages axis could be very important in neurodegeneration ALS, in which the impact of inflammation strongly contributes to the pathogenesis as reviewed in [3]. Understanding the role of EVs in the spreading of disease is essential for a deep knowledge of the signalling events occurring in ALS and involving not only microglia cells but also macrophages. This represents a clue for the exploitation of macrophage-inflamed status in therapeutic strategies for ALS. Moreover, the molecules characterizing mSOD1 NSC-34 MNs-like cell-released EVs would be of great value in the search for potential biomarkers of disease.

In conclusion, altering peripheral macrophages has the potential to regulate inflammation, impacting the progression of the disease and the survival of individuals with ALS, with timing playing a critical role in intervention.

## Figures and Tables

**Table 1:** Macrophage polarization markers expression following incubation of Raw 264.7 with CM of mSOD1 NSC-34 cells.

		*A4V*	*G37R*	*G85R*	*G93A*
Pro-inflammatory macrophage markers	NFkB	+1.69	+4.83	+3.75	+2.41
	TNFα	=	=	=	=
	IL-1β	=	+2.75	=	+4.23
	IFNα	+2.42	+2.81	+2.25	=
	IL-6	+1.91	+2.46	+2.35	=
Anti-inflammatory macrophage markers	IL-10	=	+1.89	+3.62	+3.83
	TGFβ	+1.84	+6.59	+4.79	+3.84
	IL-4	+1.75	=	+2.4	+1.8

## Data Availability

The data that support the findings of this study are available upon request to the corresponding author.
